# *Codonopsis lanceolata* Extract Restores Smooth Muscle Vasorelaxation in Rat Carotid Arteries Even under High Extracellular K^+^ Concentrations

**DOI:** 10.3390/nu15173791

**Published:** 2023-08-30

**Authors:** Uihwan Kim, You Kyoung Shin, Jubin Park, Geun Hee Seol

**Affiliations:** 1 Department of Basic Nursing Science, College of Nursing, Korea University, Seoul 02841, Republic of Korea; 2 FOUR Program of Transdisciplinary Major in Learning Health Systems, Graduate School, Korea University, Seoul 02841, Republic of Korea

**Keywords:** *Codonopsis lanceolata*, endothelial dysfunction, potassium, vasorelaxation, voltage-dependent Ca^2+^ channel

## Abstract

Recent studies showed that *Codonopsis lanceolata* (CL) has antihypertensive effects. However, to date, no study has examined the effects of CL on vascular tone under a high extracellular K^+^ concentration ([K^+^]_o_). Thus, the present study examined the effect of an extract of *Codonopsis lanceolata* (ECL) on the vascular tension of rat carotid arteries exposed to high [K^+^]_o_. We used myography to investigate the effect of an ECL on the vascular tension of rat carotid arteries exposed to high [K^+^]_o_ and the underlying mechanism of action. In arteries with intact endothelia, the ECL (250 μg/mL) had no effect on vascular tension in arteries exposed to normal or high [K^+^]_o_. In contrast, the ECL significantly increased vasorelaxation in endothelium-impaired arteries exposed to a physiologically normal or high [K^+^]_o_ compared with control arteries exposed to the same [K^+^]_o_ conditions in the absence of ECL. This vasorelaxing action was unaffected by a broad-spectrum K^+^ channel blocker and an ATP-sensitive K^+^ channel blocker. The ECL significantly inhibited the vasoconstriction induced by Ca^2+^ influx through voltage-dependent Ca^2+^ channels (VDCCs) but not Ca^2+^ influx induced via receptor-operated Ca^2+^ channels or the release of Ca^2+^ from the sarcoplasmic reticulum in the vascular smooth muscle. In summary, our study reveals that the ECL acts through VDCCs in vascular smooth muscle to promote the recovery of vasorelaxation even in arteries exposed to high [K^+^]_o_ in the context of endothelial dysfunction and provides further evidence of the vascular-protective effects of ECL.

## 1. Introduction

Blood vessels play an essential role in the homeostatic regulation of peripheral vascular resistance through changes in lumen diameter which, in turn, is controlled by factors that promote vascular constriction or relaxation [1]. Vascular constriction or relaxation (i.e., vascular tone) is mainly influenced by alterations in the cytosolic free calcium (Ca^2+^) concentration [2] and also by changes in membrane potential [3].

Potassium (K^+^), one of the most abundant cations in the human body, plays a vital role in maintaining the resting membrane potential of cells [4]. Because only ~2% of the total K^+^ in the human body is located extracellularly [5], the concentration of K^+^ ([K^+^]_o_) in the extracellular space can easily become elevated in varied pathological settings, such as chronic kidney disease (CKD) [6], rhabdomyolysis [7], and burns [8]. 

Vascular smooth muscle cells express various types of K^+^ channels [9]; notably, changes in [K^+^]_o_ lead to changes in the cell membrane potential that induce vasoconstriction (elevated [K^+^]_o_) or vasodilation (decreased [K^+^]_o_) [10]. Inappropriate changes in vascular function caused by uncontrolled elevations in [K^+^]_o_ can lead to secondary diseases such as systemic and pulmonary hypertension, hypercholesterolemia, and arteriosclerosis [11]. Therefore, it would be very beneficial to prevent pathological vascular function changes induced by elevated [K^+^]_o_.

*Codonopsis lanceolata* (CL), generally known as a traditional therapeutic plant, has been widely used in East Asian countries owing to its various medicinal effects, which include anti-inflammatory, antioxidant, and antimicrobial properties [12]. Various components of CL contribute to its medicinal effects, including saponins, polyphenols, tannins, and flavonoids [13]. For instance, lancemaside A and echinocystic acid, saponin components of an extract of *Codnopsis lanceolata* (ECL) and its metabolite, respectively, relieved 2, 4, 6-trinitrobenzenesulfonic acid-induced colitis mice [14,15]. Also, total polysaccharides, which are crucial bioactive compounds in ECL, ameliorated insulin resistance in mice fed a high-fat/high-fructose diet via anti-oxidative actions [16].

Furthermore, recent studies have suggested that an ECL and its components can also improve vascular function [17,18,19,20]. Specifically, an ECL was shown to significantly decrease systolic blood pressure in both a rat model of hypertension and in prehypertensive adults [19,20]. Some studies demonstrated that a saponin component of an ECL improves endothelial cell function by activating endothelial nitric oxide synthase [17] and modulating nitric oxide bioavailability in hypertensive rat aorta tissue [18]. Based on the previous studies, it is likely that that Ca^2+^ channels are involved in the working mechanism of CL, similar to the case of nifedipine, a well-known Ca^2+^ channel blocker that functions by inhibiting intracellular Ca^2+^ influx into the blood vessels and heart. For example, an ECL contributed to Ca^2+^ homeostasis in vascular endothelial and smooth muscle cells [21]. Also, an ECL showed a significant hypotensive effect in a hypertensive rat model, although not to the same degree as nifedipine [19]. 

In short, no previous study has examined how an ECL affects vascular tone among rat carotid arteries exposed to high [K^+^]_o_. Accordingly, we sought to investigate how an ECL affects changes in the vascular tension of rat carotid arteries that are exposed to high [K^+^]_o_ and to determine the possible underlying mechanism of this action.

## 2. Materials and Methods

### 2.1. Preparation of Plant Extracts and Chemicals 

An extract of *Codonopsis lanceolata* (ECL) was prepared as previously described [19]. Briefly, sliced CL roots obtained from Panax Korea were lyophilized and extracted at 60 °C for 270 min, using 55% ethanol using a condenser. The extract was filtered (Whatman Inc., Clifton, NJ, USA) to remove undissolved residue, concentrated using a rotary evaporator (N-1000S; EYELA, Tokyo, Japan), and lyophilized into a powder. Phenylephrine (PE), acetylcholine (ACh), Nω-nitro-L-arginine methyl ester (L-NAME), potassium chloride (KCl), calcium chloride (CaCl_2_), ethylene glycol-bis (β-aminoethyl ether)-N,N,N′,N′-tetraacetic acid (EGTA), tetraethylammonium (TEA), and glibenclamide (GLB) were purchased from Sigma Aldrich (St. Louis, MO, USA). All chemicals were dissolved in distilled water with the exception of GLB, which was dissolved in dimethyl sulfoxide.

### 2.2. Experimental Animals and Myography

Male 3-week-old Sprague Dawley rats (72.79 ± 0.65 g) from YoungBio (Seongnam, Republic of Korea) were used in these experiments. The rats were acclimatized to their environment for 1 week prior to the initiation of the experiment. The rats were 4 weeks old after the acclimatization period. All the rats were raised in standard environmental conditions (a 12 h dark/light cycle at 22 ± 1 °C with lights on/off at 08:00/20:00 and 50 ± 5% humidity), with standard chow and tap water provided ad libitum. The rats were anesthetized with isoflurane and sacrificed, after which their carotid arteries were carefully dissected and cut into rings measuring 2–3 mm in length. The vessel rings were then mounted on thin tungsten wires in an organ chamber (DMT620M; Aarhus, Denmark) filled with Krebs solution (118.3 mM of NaCl, 4.78 mM of KCl, 25 mM of NaHCO_3_, 1.22 mM of KH_2_PO_4_, 11.1 mM of glucose, 2.5 mM of CaCl_2_·2H_2_O, and 1.2 mM if MgCl_2_·6H_2_O) and maintained at 37 °C with continuous aeration (95% O_2_ and 5% CO_2_). The resting tension was set to 1 g for 60 min. After the resting tension of the carotid arteries became stable, PE (10 μM) was added to evoke maximum vasoconstriction under normal [K^+^]_o_ conditions. Vasoconstriction and vasorelaxation were confirmed via the sequential administration of 12 mM or 18 mM of KCl (high [K^+^]_o_, [22]) and ACh. Vasorelaxation was calculated as a percentage relative to that which caused maximum vasoconstriction. The vessel rings were pretreated with 50 μg/mL or 250 μg/mL of an ECL (ECL50 and ECL250, respectively) or 100 of μM L-NAME 30 min before the addition of PE. All procedures were carried out in accordance with the institutional guidelines for the use and care of laboratory animals and were approved by the Ethics Committee of Korea University (Protocol Number: KUIACUC-2021-0088).

### 2.3. Involvements of Extracellular Ca^2+^ Influx and Intracellular Ca^2+^ Release in the Effects of ECL on Vascular Tension

To investigate whether the ECL mediates changes in intracellular Ca^2+^ via Ca^2+^ channels in vascular smooth muscle cells, we evaluated two Ca^2+^ channels—voltage-dependent Ca^2+^ channels (VDCCs) and receptor-operated Ca^2+^ channels (ROCCs)—using different protocols [23]. Changes in vasoconstriction mediated by VDCCs or ROCCs were monitored after the addition of CaCl_2_ (10^−6^ to 10^−2^ M) to endothelium-denuded rat carotid artery rings which were pre-constricted with 60 mM of KCl or 10 μM of PE to promote Ca^2+^ influx via VDCCs or ROCCs in a Ca^2+^-free Krebs solution containing 10^−3^ M of EGTA. For experiments assessing the role of the endothelium, endothelial function was crippled by inhibiting the production of the major endothelium-derived vasodilator, nitric oxide (NO), using 100 μM of L-NAME, which was included in the bath solutions. After washing the vessel rings three times, they were pretreated with the ECL (250 μg/mL), followed by addition of KCl or PE. The vasoconstrictions induced by CaCl_2_ were calculated relative to those induced by 10^−2^ M CaCl_2_ in the absence of ECL, defined as 100% [24]. 

To assess whether the ECL was involved in intracellular Ca^2+^ release, we indirectly evaluated the release of Ca^2+^ from the sarcoplasmic reticulum (SR) by comparing the two PE-induced constrictions. Carotid artery rings were incubated in a Ca^2+^-free Krebs solution containing 100 μM of L-NAME and 10^−3^ M of EGTA. After first monitoring the PE (10 μM)-induced vasoconstriction for 20 min, the vessels were washed three times with a normal Krebs solution and then pretreated with the ECL (250 μg/mL) for 15 min. Thereafter, PE was again added, and the second PE-induced vasoconstriction was monitored [24].

### 2.4. Involvement of K^+^ Channels in the Effects of ECL on Vascular Tension

To identify K^+^ channels that might be involved in mediating the effect of the ECL, we used two K^+^ channel blockers [24], the broad-spectrum K^+^ channel blocker TEA (1 mM) and the ATP-sensitive K^+^ (K_ATP_) channel blocker GLB (1 μM). In myography experiments (described above), each of these K^+^ channel-blocking agents was added 10 min before the addition of PE.

### 2.5. Statistical Analysis

Statistical analyses were performed using SPSS statistics version 23.0 software (IBM, Chicago, IL, USA). Data are presented as means ± the standard error of the mean (SEM). Differences between two groups were analyzed using paired *t*-tests, and differences among multiple groups were analyzed via a one-way analysis of variance (ANOVA) followed by Tukey’s post hoc test. Statistical significance was accepted for *p*-values < 0.05.

## 3. Results

### 3.1. Effects of ECL on Vascular Tension According to [K^+^]_o_

**Figure 1:**
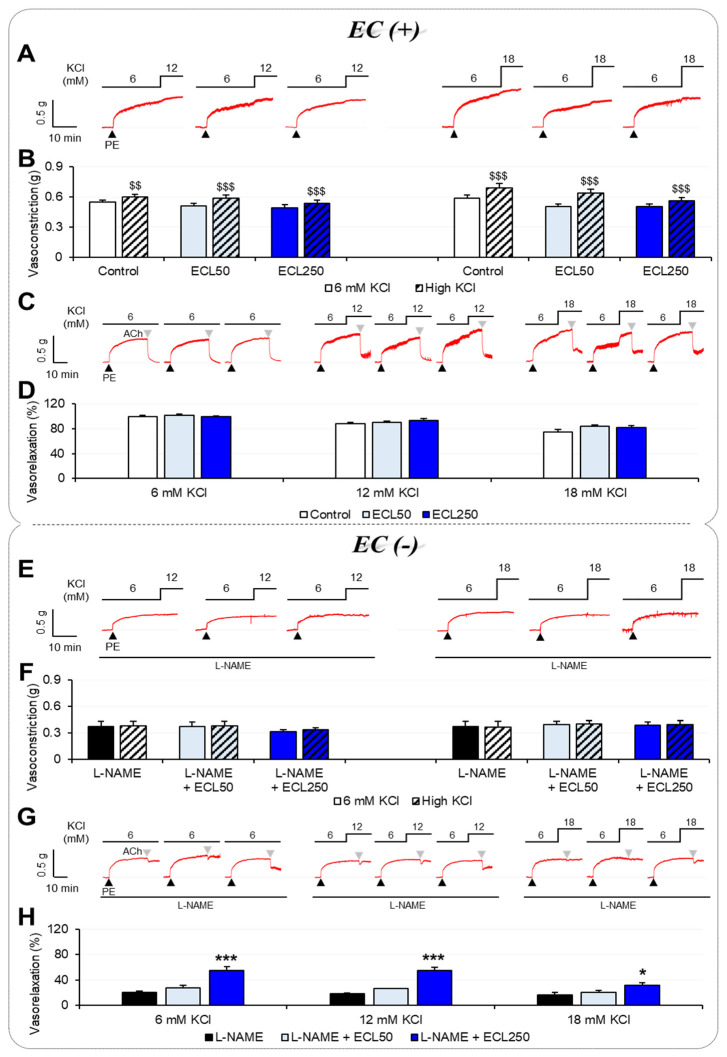
Effects of an ECL on the vascular tension of rat carotid arteries when exposed to normal or high [K^+^]_o_. Experimental traces with difference of PE-induced vasoconstriction (**A**,**B**) and ACh-induced vasorelaxation (**C**,**D**) when exposed to normal or high [K^+^]_o_ (12 or 18 mM) in carotid arteries with intact endothelia. Experimental traces with differences in PE-induced vasoconstriction (**E**,**F**) and ACh-induced vasorelaxation (**G**,**H**) when exposed to normal or high [K^+^]_o_ in endothelium-impaired carotid arteries. Results are displayed as means ± standard error of the mean and were analyzed via a paired *t*-test or one-way ANOVA followed by Tukey’s post hoc test (*n* = 5 to 22). ^$$^ *p* < 0.01, ^$$$^ *p* < 0.001 respective to 6 mM of KCl; * *p* < 0.05, *** *p* < 0.001 versus L-NAME. ACh, acetylcholine; EC, endothelial cell; ECL, extract of *Codonopsis lanceolata*; L-NAME, Nω-nitro-L-arginine methyl ester; PE, phenylephrine.

Changes in vasoconstriction and vasorelaxation in the rat carotid arteries with intact endothelia as [K^+^]_o_ was increased from normal to high KCl are displayed in Figure 1A–D. When [K^+^]_o_ increased from 6 mM to 12 mM KCl, the K^+^ alone (control group) demonstrated significantly increased vasoconstriction, measured as vascular tension (0.55 ± 0.02 g to 0.60 ± 0.03 g; *n* = 11, *p* = 0.002), as well as when added to the ECL50 group (0.51 ± 0.03 g to 0.59 ± 0.03 g; *n* = 15, *p* < 0.001) or ECL250 group (0.49 ± 0.03 g to 0.53 ± 0.03 g; *n* = 15, *p* < 0.001). Similarly, K^+^-dependent vasoconstriction was significantly elevated in the control group (0.59 ± 0.03 g to 0.69 ± 0.04 g; *n* = 10, *p* < 0.001), ECL50 group (0.50 ± 0.03 g to 0.63 ± 0.04 g; *n* = 22, *p* < 0.001), and ECL250 group (0.50 ± 0.03 g to 0.56 ± 0.03 g; *n* = 17, *p* < 0.001) as [K^+^]_o_ increased from 6 mM to 18 mM KCl (Figure 1A,B). Although increasing [K^+^]_o_ from 6 mM to 18 mM had no significant effect on vasoconstriction, there was a trend toward a reduction in K^+^-induced vasoconstriction in the ECL250 group compared with the control group. Figure 1C,D display the different degrees of vasorelaxation observed with 6 mM of KCl, 12 mM of KCl, and 18 mM of KCl. Vasorelaxation was not significantly different among the vessels of the control group with intact endothelia, ECL50 group, and ECL250 group in the presence of 6 mM of KCl, 12 mM of KCl, or 18 mM of KCl.

Figure 1E,H show changes in vasoconstriction and vasorelaxation in endothelium-impaired arteries as [K^+^]_o_ was increased from normal to high levels of KCl. In contrast to the arteries with intact endothelia, in the presence of L-NAME (the L-NAME group, L-NAME + ECL50 group, and L-NAME + ECL250 group), K^+^ failed to increase vasoconstriction. There was no difference in vasoconstriction between 6 mM and 12 mM or 6 mM and 18 mM of KCl in the L-NAME group, L-NAME + ECL50 group, or L-NAME + ECL250 group (Figure 1E,F). In endothelium-impaired vascular beds exposed to 6 mM of KCl plus the L-NAME-induced disruption of NO production, vasorelaxation was significantly increased in the L-NAME + ECL250 group (54.96 ± 5.56%; *n* = 6, *p* < 0.001) compared to the L-NAME group (20.20 ± 2.06%; *n* = 6). This phenomenon in the L-NAME + ECL250 group was also observed even when [K^+^]_o_ was elevated to 12 mM (55.40 ± 5.02%; *n* = 6, *p* < 0.001) or 18 mM of KCl (31.85 ± 4.10%; *n* = 6, *p* = 0.027) compared to the L-NAME group (18.15 ± 1.39%; *n* = 5, 16.50 ± 3.84%; *n* = 6) (Figure 1G,H).

### 3.2. Involvement of Ca^2+^ Influx or Release in the Effect of ECL

**Figure 2:**
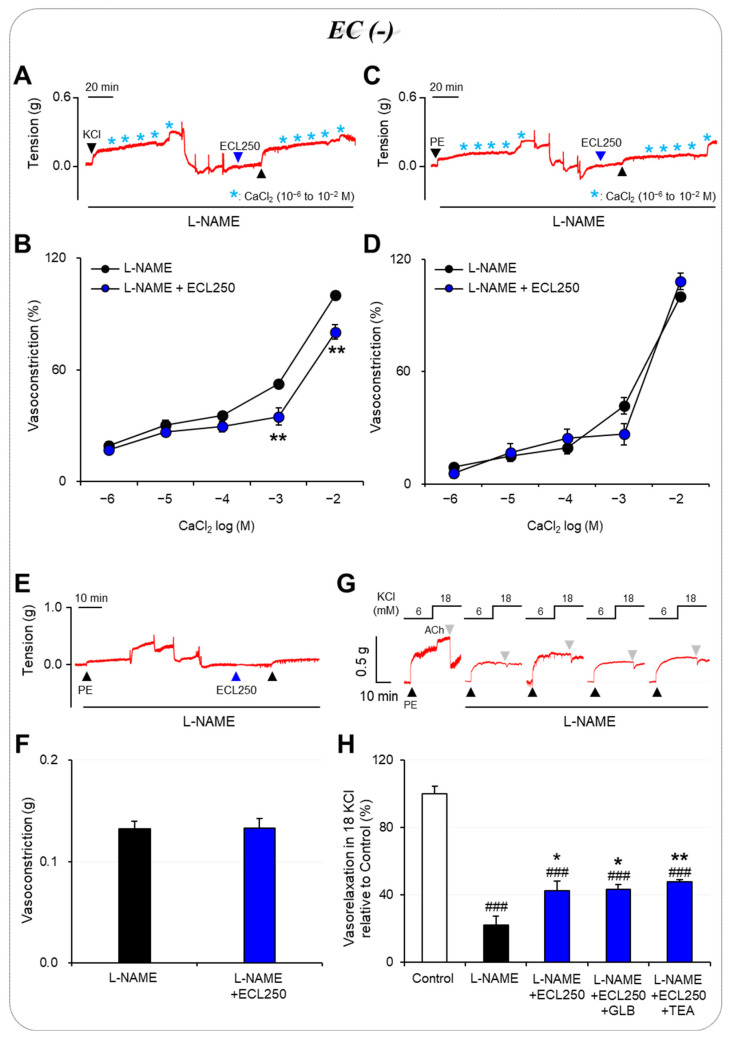
Underlying mechanism of action of ECL in vascular smooth muscle. Extracellular Ca^2+^ influx via voltage-dependent Ca^2+^ channel (**A**,**B**). Extracellular Ca^2+^ influx via receptor-operated Ca^2+^ channel (**C**,**D**). Intracellular Ca^2+^ release from sarcoplasmic reticulum (**E**,**F**). Activation of non-selective K^+^ channel or ATP-sensitive K^+^ channel (**G**,**H**). Results are expressed as means ± standard error of the mean. Analysis was performed via one-way ANOVA followed by Tukey’s post hoc or paired *t*-test (*n* = 5 to 13). ^###^ *p* < 0.001 vs. Control; * *p* < 0.05, ** *p* < 0.01 vs. L-NAME. ECL, extract of *Codonopsis lanceolata*; GLB, glibenclamide; TEA, tetraethylammonium; L-NAME, Nω-nitro-L-arginine methyl ester; PE, phenylephrine.

To determine whether the effect of the ECL is related to Ca^2+^ influx, we investigated VDCCs by using 60 mM of KCl and ROCCs by using 10 μM of PE. These experiments showed that pretreatment with ECL significantly reduced CaCl_2_-induced vasoconstriction in Ca^2+^-free solutions compared with the control group only under conditions in which the vessel was pre-constricted with KCl (*p* < 0.01; Figure 2A–D). This suggests that the ECL does not block Ca^2+^ influx via ROCCs but instead blocks VDCCs. To test whether the ECL affects vascular tone through the interruption of the release of Ca^2+^ from the SR, we measured PE-induced vasoconstriction under Ca^2+^-free conditions. A comparison of vasoconstriction in the presence and absence of the ECL showed no significant difference (Figure 2E,F), indicating that the mechanism through which the ECL regulates vascular tone is not affected by the release of Ca^2+^ from the SR. 

### 3.3. Involvement of K^+^ Channels in the Effect of ECL

Moreover, pretreatment with TEA (1 mM) or GLB (1 μM), which are broad-spectrum K^+^ channel and K_ATP_ channel blockers, respectively, did not affect the recovery of ECL-induced vasorelaxation in the L-NAME–pretreated carotid arteries exposed to 18 mM of KCl (Figure 2G,H). These results imply that neither the broad range of K^+^ channels inhibited by TEA nor the K_ATP_ channels in vascular smooth muscle cells are involved in mediating the effects of ECL under high [K^+^]_o_ conditions.

## 4. Discussion

**Figure 3:**
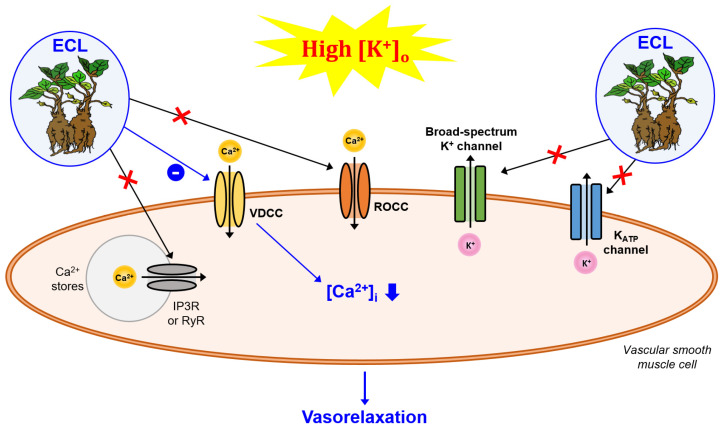
Mechanism underlying the vasorelaxant effects of ECL on the vascular smooth muscle of rat carotid arteries exposed to high [K^+^]_o_. ECL, extract of *Codonopsis lanceolata*; IP3R, inositol 1,4,5-trisphosphate receptor; ROCC, receptor-operated Ca^2+^ channel; RyR, ryanodine receptor; VDCC, voltage-dependent Ca^2+^ channel; [Ca^2+^]_i_, intracellular Ca^2+^ concentration; [K^+^]_o_, extracellular K^+^ concentration.

The purpose of this study was to investigate the effect of the ECL on changes in the vascular tone of rat carotid arteries following exposure to high [K^+^]_o_ and to determine the possible underlying mechanism of action. In the context of endothelial dysfunction, vessel rings pretreated with 250 μg/mL of ECL showed significantly more vasorelaxation than control rings, even under high [K^+^]_o_. Our results suggest that ECL contributes to improving the vasorelaxant responses of carotid arteries in an endothelium-independent manner, even in the presence of high [K^+^]_o_. VDCC, not ROCC, the release of Ca^2+^ from intracellular Ca^2+^ stores, K_ATP_ channels, and a broad range of K^+^ channels were involved in mediating the effects of ECL under high [K^+^]_o_ conditions (Figure 3).

The endothelium contributes to vasomotor homeostasis by releasing substances that are important in vasorelaxation, primarily NO and endothelium-derived relaxing factors. Therefore, endothelial dysfunction disables important vasorelaxation mechanisms, leading to impaired vasomotor homeostasis. Alterations in the vasomotor properties of peripheral blood vessels can influence the regulatory functions controlling peripheral blood vessel resistance, thereby affecting proper blood pressure control. Numerous studies have highlighted the pivotal role of the endothelium in the effect of ECL on blood vessels. For instance, lancemaside A enhances endothelial function through the activation of endothelial NO synthase [17] and prevents hypertension by improving NO bioavailability in rats [18]. In a separate study by Han et al. (2018), an ECL was shown to have a significant blood pressure-reducing effect and tended to increase ACh- and SNP-induced vasorelaxation in a hypertensive rat model [19]. However, our current findings demonstrate that, despite endothelial dysfunction, the ECL exerted a prominent vasorelaxing effect even in carotid arteries exposed to high [K^+^]_o_, countering the vasoconstricting effect of this latter condition. 

The Ca^2+^ channel-mediated alteration of intracellular Ca^2+^ concentrations in both endothelial and vascular smooth muscle cells is important for vasomotion. Vascular smooth muscle cells express various types of Ca^2+^ channels that play important roles in controlling vascular tone [25]. Here, we evaluated two such channels expressed in smooth muscle, namely, VDCCs and ROCCs, for their involvement in the ability of the ECL to modulate vascular tone. High [K^+^]_o_ promotes Ca^2+^ influx through VDCCs, whereas PE-mediated α-receptor activation promotes Ca^2+^ influx through ROCCs [24]. Therefore, we estimated the CaCl_2_-induced vasoconstrictive responses of the arteries that had been pre-constricted via KCl or PE in a Ca^2+^-free solution containing EGTA. Our results showed that the ECL did not significantly change vasoconstriction induced by Ca^2+^ influx through ROCCs but significantly reduced vasoconstriction induced by Ca^2+^ influx through VDCCs. We also evaluated the involvement of the release of Ca^2+^ from intracellular Ca^2+^ stores and found no significant difference in vasoconstriction induced by Ca^2+^ release from the SR following pretreatment with the ECL. Collectively, these findings suggest that one of the mechanisms through which ECL improves vasorelaxation is through the inhibition of Ca^2+^ influx via VDCCs in vascular smooth muscle. In a previous study, the oral administration of an ECL (200 or 400 mg/kg) in a rat model of hypertension rats had less of an effect compared with the administration of the L-type Ca^2+^ channel blocker nifedipine, but it nonetheless significantly reduced systolic blood pressure compared with rats in a vehicle control group [19]. Moreover, lancemaside A, a major saponin component of an ECL, contributed to Ca^2+^ homeostasis by inhibiting Ca^2+^ influx through store-operated Ca^2+^ entry in vascular smooth muscle cells [21], which suggests that effects of the ECL in this study could be induced by lancemaside A. Increased [K^+^]_o_ is a crucial factor that can cause changes in vascular tone [26]. Specifically, high [K^+^]_o_ causes the depolarization of the cell membrane, which activates VDCCs, increasing Ca^2+^ influx and promoting the Ca^2+^-dependent vasoconstriction of vascular smooth muscle [27,28]. Our results collectively suggest that the ECL contributes to improving impaired vasorelaxant responses by inhibiting the ability of VDCCs in vascular smooth muscle to respond to the vasoconstrictive effects of high [K^+^]_o_. 

Various pathophysiological situations can cause endothelial dysfunction; some of them also carry a risk of elevated [K^+^]_o_. For example, rhabdomyolysis was reported to induce damage to endothelial cells in mice [29], and simultaneous cell death due to rhabdomyolysis can acutely increase [K^+^]_o_ because most K^+^ ions are contained within the intracellular compartment [30,31]. Similarly, CKD patients are vulnerable to [K^+^]_o_ elevation due to deterioration of the kidney’s K^+^ excretion function [6]. Numerous factors in CKD can impair the bioavailability of nitric oxide [32], and CKD-related cardiovascular disease is primary based on endothelial dysfunction [33]. In this context, our results demonstrate that the ECL significantly improves vascular vasorelaxant responses caused by endothelial dysfunction under elevated [K^+^]_o_.

Here, we found that the ECL effectively restores impaired vasorelaxation caused by endothelial dysfunction even when exposed to high [K^+^]_o_. Previous studies have suggested that most of the beneficial effects of an ECL or a major saponin of CL on blood vessels apply only in pathological situations, such as in a hypertensive rat model [18,19]. Interestingly, however, our present results showed that the ECL improved the vasorelaxation induced by endothelial dysfunction under elevated [K^+^]_o_ and it did not negatively affect vascular tone under normal physiological [K^+^]_o_ or when the endothelium remained intact. 

## 5. Conclusions

In conclusion, this study showed for the first time that an ECL significantly restored of smooth muscle vasorelaxation in endothelium-impaired rat carotid arteries even under high [K^+^]_o_. The present results add to the existing knowledge of the beneficial properties of ECLs on vascular function and indicate that an ECL could be developed as an alternative medicinal agent in patients with elevated [K^+^]_o_ who are at risk for endothelial dysfunction. Because the present experiments were only conducted on the carotid arteries of rats, further clinical research on the safety and efficacy of using an ECL in humans is needed.

## Data Availability

Data are available upon reasonable request from the corresponding author.
